# Supplementary material for: Time-synthetic optical neural networks with stable programmable gain
Source: Nat Commun. 2026 May 6;17:6068. doi: 10.1038/s41467-026-72773-2 (PMC13350085; doi:10.1038/s41467-026-72773-2)
Supplement: Supplementary file 1 — Supplementary Information [file 41467_2026_72773_MOESM1_ESM.pdf]

Supplementary Information for

Time-synthetic optical neural networks with stable programmable gain

Bei Wu, Yudong Ren, Rui Zhao, Haiyao Luo, Fujia Chen, Li Zhang, Lu Zhang, Hongsheng Chen* and Yihao Yang*

[1] *State Key Laboratory of Extreme Photonics and Instrumentation, ZJU-Hangzhou Global Scientific and Technological Innovation Center, Zhejiang University, Hangzhou 310027, China.*

[2] *International Joint Innovation Center, The Electromagnetics Academy at Zhejiang University, Zhejiang University, Haining 314400, China.*

[3] *Key Lab. of Advanced Micro/Nano Electronic Devices & Smart Systems of Zhejiang, Jinhua Institute of Zhejiang University, Zhejiang University, Jinhua 321099, China.*

[4] *Shaoxing Institute of Zhejiang University, Zhejiang University, Shaoxing 312000, China.*

* *Corresponding authors: hansomchen@zju.edu.cn (H. Chen); yangyihao@zju.edu.cn (Y. Yang)*

The PDF file includes:

Supplementary Note 1: Quantitative analysis of fidelity

Supplementary Note 2: Implementation of 10 × 10 matrix

Supplementary Note 3: Implementation of programmable gain/loss

Supplementary Note 4: Elimination of parasitic reflections

Supplementary Note 5: Case of fully loaded coupled loops

Supplementary Note 6: Time-synthetic ONN’s operation speed

Supplementary Note 7: Proposal of an integrated time-synthetic ONN

Supplementary Note 8: Experimental signal monitoring and propagation-field reconstruction

Supplementary Note 1: Quantitative analysis of fidelity

To quantitatively analyse the impact of noise on fidelity, we conduct experiments using the same configuration as in the image classification tasks. Specifically, a square pulse with >50 dB extinction ratio and 50 ns temporal width is injected into the longer loop. The variable BS splits each pulse with a predefined beam-splitting ratio of 50:50, and the shorter loop integrates an MZM for dynamic gain/loss modulation.

To evaluate the repeatability and stability of the measurements, we perform repeated measurements under a fixed gain/loss configuration and calculate the fidelity for up to 110 round trips using the formula $F = (1 - |\hat{Y} - Y|/(\hat{Y} + Y)) \times 100\%$, where Y is the optical signal without distortion at each round trip, and $\hat{Y}$ is the experimentally measured optical signals.

The measured results are shown in Supplementary Fig. 1. The blue solid line denotes the median across repeated measurements, and the shaded region denotes the standard deviation. The experimental results show that the fidelity gradually decreases with the number of round trips, but remains sufficiently high (88.9%) over the range used in our experiments (40 round trips). After 110 round trips, the fidelity remains at 75.9%, which exceeds a commonly used engineering threshold of 67% (corresponding to an SNR of 3 dB), indicating that the time-synthetic ONN can still operate effectively with the assistance of in-situ training.

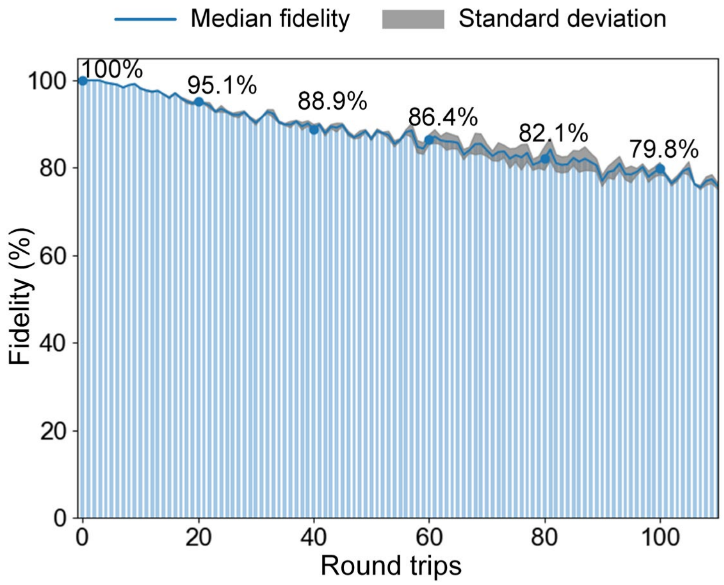

Supplementary Fig. 1 | The fidelity at different round trips.

Supplementary Note 2: Implementation of 10 × 10 matrix

The 10 × 10 matrix in Fig. 4e represents the linear transformation to be implemented by the time-

synthetic ONNs, as shown in Supplementary Fig. 2. The input-output relation is given by $O = TI$, where I and O are the complex amplitude vectors of the ten input and the ten output pulses, respectively. According to the modified discrete quantum walk equations that govern pulse propagation, the matrix T is a function of the gain/loss array:

$$T = W^0 W^1 W^2 W^3 W^4 \tag{1}$$

$$W^n = \begin{bmatrix} -G_0^n/\sqrt{2} & iG_0^n/\sqrt{2} & 0 & 0 & 0 & 0 & 0 & 0 & 0 & 0 \\ iG_5^n/2 & -G_5^n/2 & iG_1^nG_5^n/2 & G_1^nG_5^n/2 & 0 & 0 & 0 & 0 & 0 & 0 \\ 1/2 & i/2 & -G_1^n/2 & iG_1^n/2 & 0 & 0 & 0 & 0 & 0 & 0 \\ 0 & 0 & iG_6^n/2 & -G_6^n/2 & iG_2^nG_6^n/2 & G_2^nG_6^n/2 & 0 & 0 & 0 & 0 \\ 0 & 0 & 1/2 & i/2 & -G_2^n/2 & iG_2^n/2 & 0 & 0 & 0 & 0 \\ 0 & 0 & 0 & 0 & iG_7^n/2 & -G_7^n/2 & iG_3^nG_7^n/2 & G_3^nG_7^n/2 & 0 & 0 \\ 0 & 0 & 0 & 0 & 1/2 & i/2 & -G_3^n/2 & iG_3^n/2 & 0 & 0 \\ 0 & 0 & 0 & 0 & 0 & 0 & iG_8^n/2 & -G_8^n/2 & iG_4^nG_8^n/2 & G_4^nG_8^n/2 \\ 0 & 0 & 0 & 0 & 0 & 0 & 1/2 & i/2 & -G_4^n/2 & iG_4^n/2 \\ 0 & 0 & 0 & 0 & 0 & 0 & 0 & 0 & i/\sqrt{2} & -1/\sqrt{2} \end{bmatrix} \tag{2}$$

To compare the in-silico and in-situ fidelity, we randomly generated different gain/loss configurations. For each configuration, the target matrix is calculated using the ideal theoretical model (Eqs. 1-2). To obtain the experimentally realized matrices, the same gain/loss configurations are applied to the physical system. Unit-amplitude pulses are injected one at a time into the ten input ports (with the other nine ports receiving zero input), and the measured complex amplitudes at the ten output ports form the columns of the realized matrix. This procedure is repeated for all randomly generated gain/loss configurations, and the resulting fidelity yields the value reported in Fig. 4e.

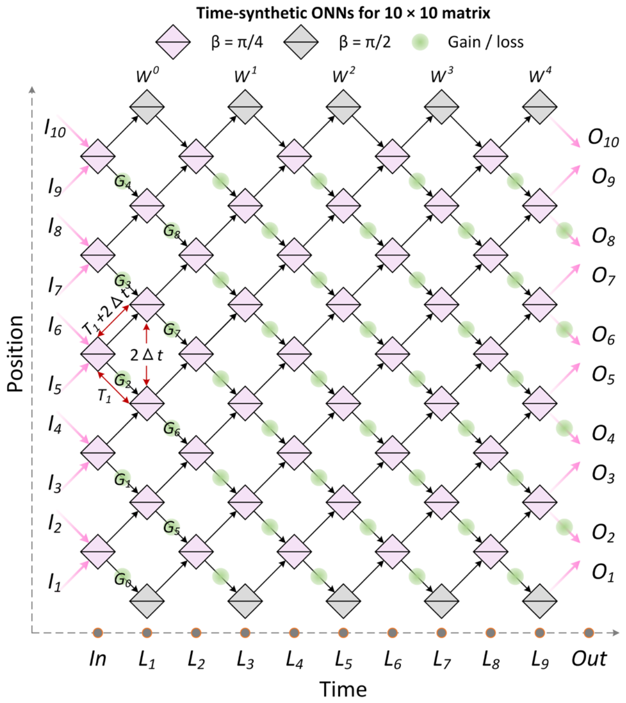

Supplementary Fig. 2 | The architecture of time-synthetic ONNs for 10 × 10 matrix representation. The splitting ratio at the boundary is set to $\beta = \pi/2$, such that the pulses are totally reflected.

Supplementary Note 3: Implementation of programmable gain/loss

In our platform, the key to achieving programmable gain/loss is the co-modulation of MZM and EDFAs. Individually, the MZM acts as a loss component. We first set the MZM modulation voltage to 1.5 V and then adjust the gains of EDFAs in the longer and the shorter loops until the measured propagation fields match the theoretical prediction for a unity-gain system, as shown in Supplementary Fig. 3a and 3b. This calibration ensures that, at 1.5 V the total loss is balanced by the EDFA gain.

After the unity-gain baseline is established, we sweep the MZM modulation voltage and measure the output amplitude relative to the unity-gain state. As shown in Supplementary Fig. 3c, this procedure allows us to span the target gain/loss range of $[e^{-0.3}, e^{0.3}]$ used in our experiments. In this way, the gain regime is realized when the MZM loss is reduced relative to the reference state, and the loss regime is realized when the MZM loss is increased relative to that state.

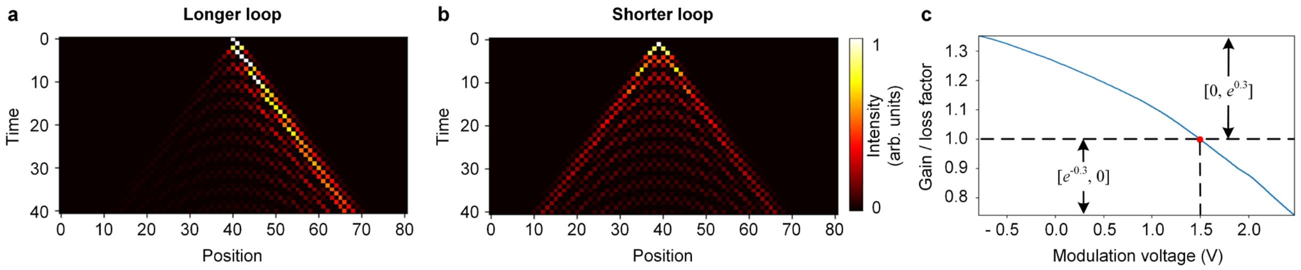

Supplementary Fig. 3 | The propagation field in **a** the longer and **b** the shorter loop in a unit-gain system. **c** The curve of the gain/loss factor as a function of the MZM modulation voltage, where 1.5 V corresponds to the unit-gain state.

Supplementary Note 4: Elimination of parasitic reflections

In optical loops, the EDFAs can introduce feedback loops and unwanted parasitic reflections. In the time-synthetic ONN, these issues are easy to control because the computation proceeds via causal, forward evolution along the time-synthetic dimension: signals propagate step-by-step to subsequent time layers. Consequently, it only needs to suppress backward in two predefined loops. In practice, only two optical isolators can prevent the parasitic reflections in the coupled loops that would disrupt the intended time-synthetic computation.

Supplementary Note 5: Case of fully loaded coupled loops

In the time-synthetic ONN, the network width—defined as the number of pulses in optical loops—

increases with the network depth (defined as the number of round-trips). The waveguide should be long enough to accommodate the pulses at the last round trip, and a significant portion of the computational space is wasted, as illustrated by the dark regions in Fig. 2d. Here, we investigate a scenario where the coupled loops are fully loaded, which decouples the dependence between network width and depth. As a representative example, we consider a configuration where the longer loop accommodates four pulses and the shorter loop accommodates three (Supplementary Fig. 4a).

Under the fully loaded condition, the number of pulses per round trip remains constant. The first pulse from the shorter loop in time layer L_n couples with the last pulse from the longer loop in the subsequent time layer L_{n+1}, as depicted in Supplementary Fig. 4b. The propagation dynamics for pulses at the leading positions ($0 \le m < M$) are governed by:

$$u_m^{n+1} = G[\cos(\beta)\, u_{m+1}^n + i\sin(\beta)\, v_{m+1}^n] e^{i\varphi} \tag{3}$$

$$v_m^{n+1} = i\sin(\beta)\, u_m^n + \cos(\beta)\, v_m^n \tag{4}$$

where u_m^n /(v_m^n) represents the complex amplitude at position m and time step n in the shorter/(longer) loop, $\beta = \beta(m, n)$ represents the splitting ratio, $G \in [e^{-0.3}, e^{0.3}]$ represents the gain/loss factor, and $\varphi \in [0, 2\pi)$ represents the phase shifter.

For pulses at the last position ($m = M$), the propagation dynamics are governed by :

$$u_M^{n+1} = u_0^n \tag{5}$$

$$v_M^{n+1} = i\sin(\beta)\, u_0^{n-1} + \cos(\beta)\, v_M^n \tag{6}$$

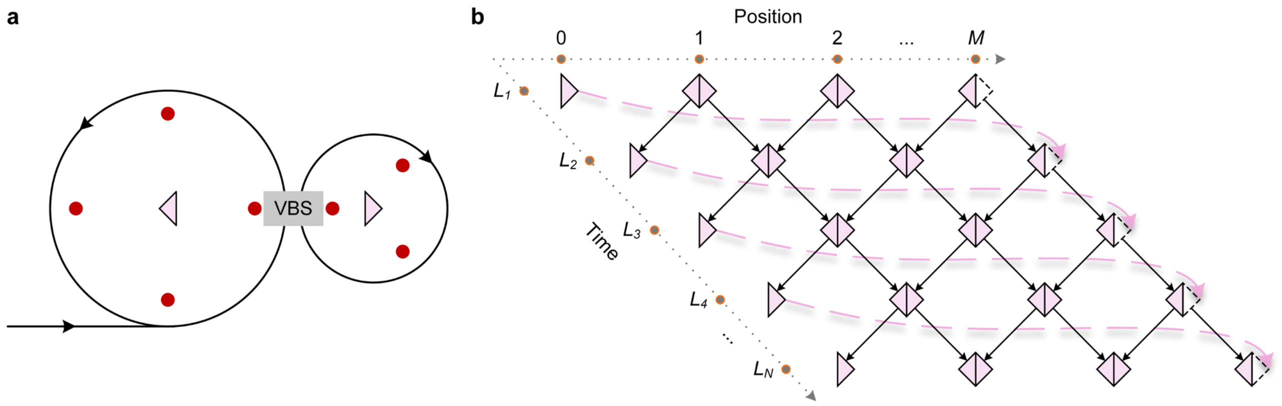

Supplementary Fig. 4 | a Fully loaded pulse propagation in coupled optical loops. **b** Time-synthetic mesh mapped from the pulse propagation in **a**.

Supplementary Note 6: Time-synthetic ONN's operation speed

In our coupled optical loops, the temporal separation between the shorter and longer loops is $2\Delta t$ = 196 ns, and the shorter-loop period is $T = 24{,}636$ ns. Under this configuration, the system can support

up to $T/\Delta t$ concurrent optical pulses. In the time-synthetic ONN, artificial neurons are sparsely connected. Considering time layers L_n and L_{n+2}, each containing $T/\Delta t$ neurons, the connections between these two layers involve $6T/\Delta t$ - 3 operations, including $4T/\Delta t$ - 2 multiplications and $2T/\Delta t$ - 1 additions.

Assuming that the optical pulses maintain high fidelity over N time layers, then within a time interval of TN, the system executes a total of $(6T/\Delta t - 3) \times N/2$ FLOPs. Therefore, with only one modulator, the operation speed of the present fiber-loop system is $R = (6T/\Delta t - 3)/2T = 30.55$ MOPS.

Importantly, we did not maximize the operational speed in the current experimental setup. The pulse-to-pulse spacing $2\Delta t$ is chosen conservatively to ensure robust operation and reliable characterization. With an optimized implementation (e.g., reducing $2\Delta t$ while maintaining stable modulation and detection), the operational speed can be increased by approximately an order of magnitude.

Supplementary Note 7: Proposal of an integrated time-synthetic ONN

While the time-synthetic ONN is experimentally validated in coupled optical loops, its architecture can be directly implemented in integrated photonic circuits, as illustrated in Supplementary Fig. 5. The experimental configuration utilizes a 1550 nm optical signal modulated into squared pulses, which are injected into the longer loop. Each loop incorporates an amplitude modulator (AM) and a PM for optical signal modulation, complemented by a semiconductor optical amplifier (SOA) to enhance signal intensity. A fraction of the light is routed to detection ports for real-time monitoring.

In the experimental setup, each loop concurrently accommodates 40 optical pulses, with an ideal separation of 10 ps between adjacent pulses. These parameters lead to a round-trip duration of approximately 0.4 ns, which can be achieved using a 3.5 cm silicon waveguide with a refractive index of 3.4. For the integrated photonic circuit operating at a wavelength of 1550 nm, waveguides are designed with a width of 450 nm and an inter-waveguide spacing of 1 μm, to mitigate crosstalk and fabrication errors. Through the optimization of bend geometries, the waveguide network enables compact integration within a die area of 181 μm × 363 μm = 0.066mm^2. The cumulative propagation time across 40 time layers is 16 ns, during which 9540 FLOPs are executed. These parameters yield a theoretical area efficiency of 13.6 Tera-FLOPs/mm^2/s (equivalent to 6.8 Tera-MACS/mm^2).

By implementing propagation loss compensation and error correction algorithms, optical pulses

undergo continuous evolution within waveguides without optoelectronic conversion. This advancement paves the way for constructing ONNs with a large number of neurons in highly compact integrated photonic circuits, highlighting the architecture's exceptional scalability for large-scale deployment.

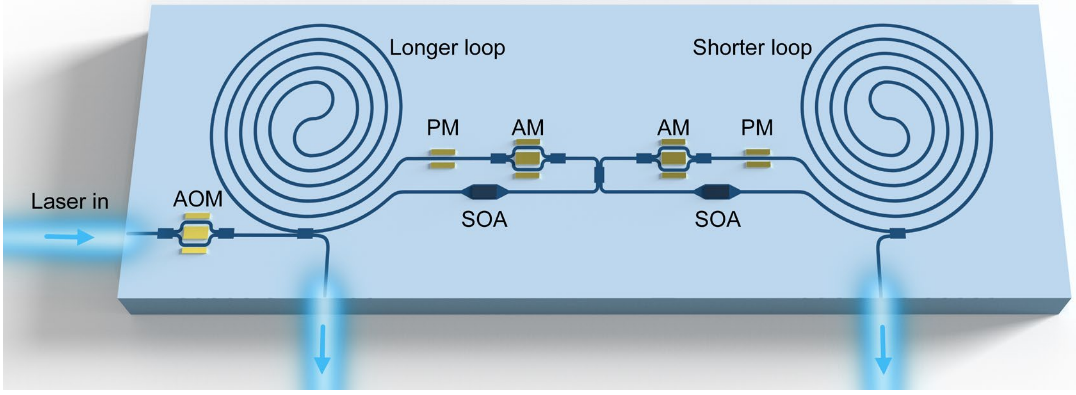

Supplementary Fig. 5 | Schematic implementation of an integrated time-synthetic ONN. AOM: acousto-optic modulator; AM: amplitude modulator; PM: phase modulator; SOA: semiconductor optical amplifier.

Supplementary Note 8: Experimental signal monitoring and propagation-field reconstruction

The optical signals in the two loops are monitored by two PDs through 50:50 BSs. As illustrated by red lines in Supplementary Fig. 6a, each BS taps half of the optical power from the loop and sends it to a PD. The PDs convert the optical signals into electronic signals, which are recorded by a DSO for further analysis.

In the monitored time-series signal, the pulse with the highest intensity is taken as the beginning position, denoted by T_l. For a pulse propagating across N round trips, the corresponding output position is $T_l + N(T + 2\Delta t)$, where $T = 24{,}636$ ns is the shorter-loop period and $2\Delta t = 196$ ns is the temporal separation between the shorter and longer loops.

The propagation field is reconstructed from the measured time-series signals. Specifically, after identifying the beginning and output positions, we extract the useful positions of the time-series signal of the longer and shorter loops; see Supplementary Fig. 6b and 6c for a 4-round-trip example. Using the known temporal relations (e.g., the shorter-loop period T and the temporal separation $2\Delta t$, the detected time-series signals are mapped onto the time-synthetic lattices. In the reconstructed propagation field, each point corresponds to a pulse, with its lower-left and lower-right neighbors separated by T and $T + 2\Delta t$, respectively, and its horizontal neighbors separated by $2\Delta t$. This procedure yields the propagation field shown in Supplementary Fig. 6d and 6e.

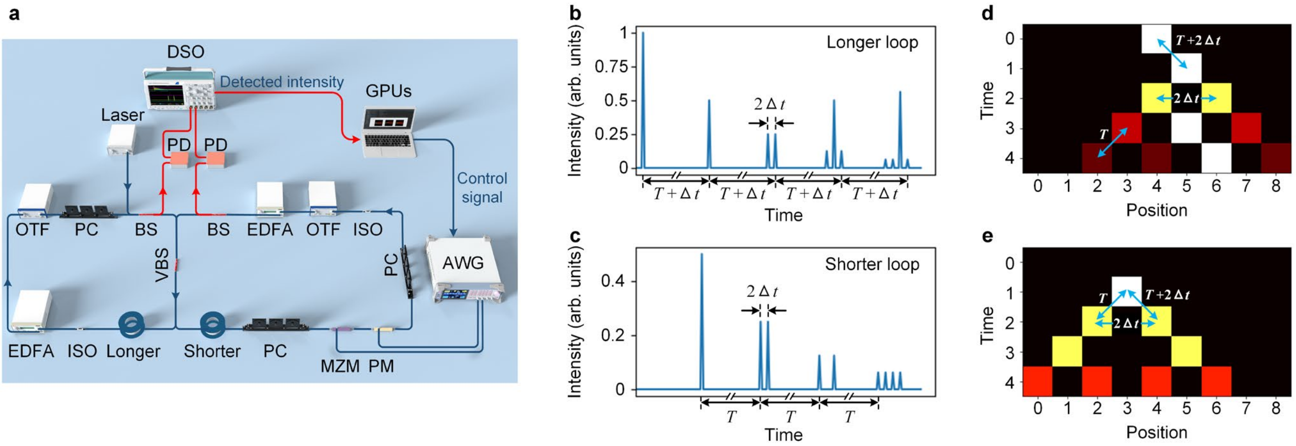

Supplementary Fig. 6 | a, The optical-loop experimental setup. Taking propagating across four round trips as an example, the time-series optical signals in **b**, the longer loop and **c**, the shorter loop. The propagation fields in **d**, the longer loop and **e**, the shorter loop.

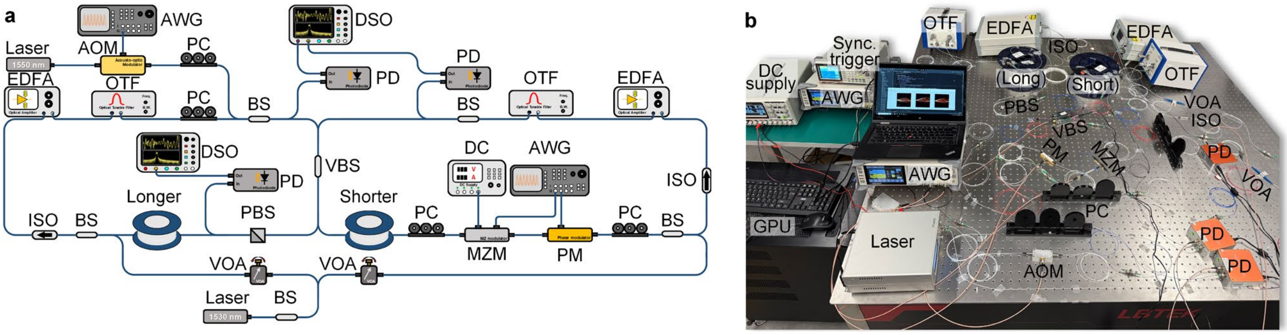

Supplementary Fig. 7 | a Experimental pulse propagation path diagram. **b** Real shot of experimental setup. AWG: arbitrary waveform generator; DSO: digital storage oscilloscope; AOM: acousto-optic modulator; PC: polarization controller; BS: beam splitter; VBS: variable beam splitter; PBS: polarizing beam splitter; PD: photodetector; EDFA: erbium-doped fiber amplifier; OTF: optical tunable filter; ISO: isolator; MZM: Mach-Zehnder modulator; PM: phase modulator; DC: direct current; VOA: variable optical attenuator.

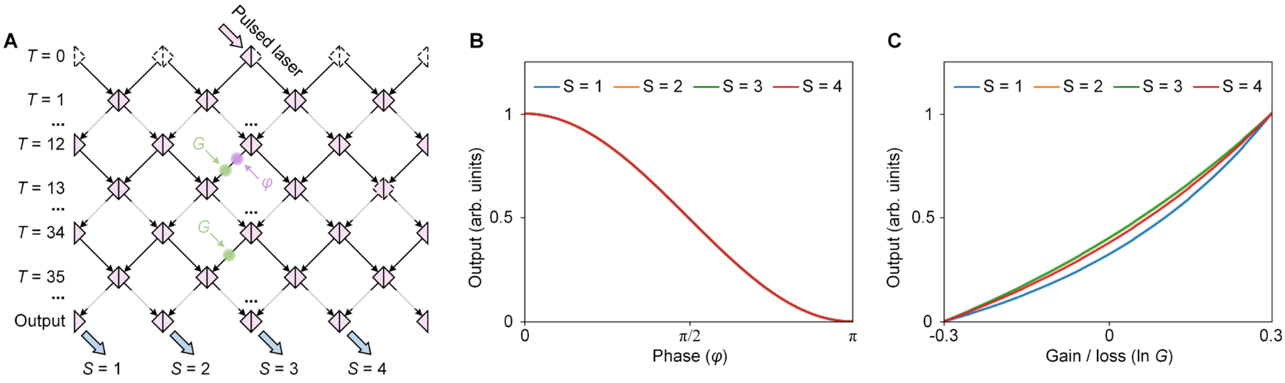

Supplementary Fig. 8 | a A time-synthetic lattice propagating through 40 round trips. **b**-**c** Output intensities at four positions in the shorter loop as a function of (**b**) the phase shifter and (**c**) the gain/loss factor, demonstrating the nonlinear input-output relationship.
