# Supplementary material for: Time-synthetic optical neural networks with stable programmable gain
Source: Nat Commun. 2026 May 6;17:6068. doi: 10.1038/s41467-026-72773-2 (PMC13350085; doi:10.1038/s41467-026-72773-2)
Supplement: Supplementary file 2 — Transparent Peer Review file [file 41467_2026_72773_MOESM2_ESM.pdf]

nature portfolio

Peer Review File

Time-synthetic optical neural networks with stable programmable gain

Corresponding Author: Professor Yihao Yang

This file contains all reviewer reports in order by version, followed by all author rebuttals in order by version.

Version 0:

Reviewer comments:

Reviewer #1

(Remarks to the Author)
The paper introduces a coupled dual-loop time-synthetic neural network platform, which can perform arbitrary complex-valued linear transformations and effectively reduce the propagation loss. In the system, the programmable gain and phase modulators were utilized to construct a non-Hermitian computational lattice. By separating network depth from the number of physical components, the architecture can simulate over 30,000 effective optical gates in a compact space and achieves remarkable scalability. The experimental results substantiate the critical role of gain, showing that with appropriate gain assistance, the network could attain high accuracy on MNIST classification, whereas without gain, performance collapses remarkably. Furthermore, the authors develop an in-situ training framework that directly derives gradients from measured optical intensities, allowing the system to adapt to hardware imperfections and noise, which enhances robustness and achieves acceptable accuracy on CIFAR-10. These contributions collectively establish gain-assisted time-synthetic ONNs as a stable, scalable, and programmable pathway toward deep photonic intelligence, effectively resolving the tension between gain necessity and instability in spatial photonic systems. The manuscript is compellingly presented through both theoretical analysis and experimental validation and is generally well-structured and clearly written. However, there are still some technical problems and details should be clarified by the authors. I would like to recommend publication of the manuscript after the following issues are addressed.
1.The manuscript employs an unconventional encoding scheme where input data is distributed across the phase modulation parameters of different time layers. Consequently, data encoded in subsequent layers interacts complexly with the signals propagated from previous layers, which appears equivalent to performing a specific pre-processing on the input data. Could the authors clarify whether this distributed encoding strategy provides a unique computational advantage, or is it simply a workaround due to the insufficient number of grid points in a single layer to accommodate all input data? A more explicit explanation is recommended.
2.The experimental setup utilizes relatively long optical fiber loops, which are inherently susceptible to environmental perturbations such as acoustic vibrations and thermal drift. Standard experiments involving long fibers typically require rigorous acoustic and thermal isolation, yet such measures appear to be absent in the described setup. Apart from relying on the in-situ training framework for error compensation, did the authors implement signal averaging over multiple measurements during data acquisition to mitigate the stochastic impact of fiber jitter?
3.I have concerns regarding the mechanism and validity of the results obtained from the in-situ training algorithm, and I request that the authors provide further clarification on the following details. It is unclear whether the in-situ training algorithm yields a single set of static compensation parameters applied globally during testing, or if it performs real-time dynamic compensation for each instance. If it is the former (static compensation), the results presented in Fig. 4e are perplexing. First, the manuscript lacks sufficient detail regarding the specific 10*10 matrix evaluated in Fig. 4e; it does not explain what this matrix represents or how the "target" and "achieved" matrices were constructed for the fidelity comparison. More critically, Fig. 4e shows that the experimental fidelity after in-situ training (98.5%) significantly exceeds the theoretical in-silico fidelity (94.8%). This is logically counter-intuitive. Theoretically, in-situ training aims to compensate for systematic errors (e.g., fabrication defects) to approach the ideal model. Therefore, the noise-free theoretical model should serve as the upper performance bound, rather than being outperformed by the experiment. If it is the latter (real-time dynamic compensation), this raises a serious concern regarding experimental design. If the algorithm utilizes the ground truth labels of the test data to calculate gradients and adjust parameters on-the-fly during the inference phase, this constitutes data leakage. Please explicitly clarify whether the one-hot encoded ground truth labels were strictly isolated from the algorithm during the inference phase to ensure no data leakage occurred.
4.I have concerns regarding the proposed neural network architecture and its reported performance. Drawing an analogy to

electronic neural networks, if each lattice point is treated as a neuron that couples only with two adjacent points in the subsequent time layer, the proposed architecture essentially functions as an extremely sparsely connected fully connected network (FCN). Theoretically, the computational capacity of such an architecture should be limited. Similar to standard FCNs, this approach appears to process images as flattened inputs, implying it lacks the inherent capability of Convolutional Neural Networks (CNNs) to effectively capture local spatial features. However, the manuscript reports an accuracy of 86.5% on the CIFAR-10 dataset, remarkably achieved after applying grayscale conversion and cropping—preprocessing steps that typically result in information loss and reduced accuracy. This result appears anomalous, as it significantly outperforms not only standard CNN benchmarks for CIFAR-10 (approximately 82%) but also certain optimized deep convolutional architectures (e.g., 84.87% reported in Stochastic Pooling for Regularization of Deep Convolutional Neural Networks). Given the structural sparsity of the network and the lossy preprocessing of the input data, such superior performance seems counter-intuitive. I request that the authors provide a rigorous explanation for this discrepancy.
5.Although the authors introduce optical gain to compensate for propagation loss, various components within the loops—such as PM, MZM, and EDFAs—inevitably introduce irregular noise, which accumulates with the number of round trips. The manuscript does not provide a quantitative analysis of this accumulated noise, nor does it clearly specify the upper limit on the number of round trips over which optical pulses can maintain sufficiently high fidelity for reliable computation.
6.In lines 226–231, the effective number of gates is estimated based on the evolution of a single pulse in the synthetic temporal lattice. However, since the number of pulses increases with each round trip, the total number of effective gates cannot be simply expressed as the product of the number of pulses accommodated by the fiber and the number of round trips. A more rigorous justification or clarification of this scaling relation is required.
7.The authors mention that the system remains stable as long as the programmed gain stays below the point where instability would occur. They should provide a detailed explanation of the theoretical methods or experimental approaches used to determine this instability threshold in synthetic temporal lattice.
8.With hundreds of pulses propagating concurrently in the loops, potential crosstalk between adjacent pulses could affect computational accuracy. The paper reports a coupled experimental platform containing over 30,000 effective time gates. What experimental or analytical evidence proves that the computational precision (e.g., the effective bit-depth or phase stability of the artificial neurons) does not degrade after, for example, 100 round trips? Furthermore, are there error correction or calibration routines embedded in the in-situ training that specifically compensate for predictable distortion accumulated over time?

Reviewer #2

(Remarks to the Author)
This work presents an experimental demonstration of optical neural networks based on coupled fiber loops, demonstrating a benefit of optical gain on the network operation. Such experimental systems, also incorporating gain elements, have been introduced and extensively investigated for over a decade. The main novelty here is the analysis of gain effect specifically for neural-network applications.

Overall, the technical quality is high, and results are generally convincing. There are some questions that need clarification:

1) The aspect of phase-modulation is not fully clear. The theoretical design incorporates the training of nontrivial phase modulation. However, in experiments, the phase modulation is absent, in order to perform experimental training just based on the output intensities. Then, why was the phase modulation included in the theory at all? Is there any specific downside to omitting the phase modulation in experiments?

2) Is there a practical limit on the number of round-trip times. It is about 40 in the current study, but what is a practical limit, and how does the gain and noise influence it?

Reviewer #3

(Remarks to the Author)
Wu et al. demonstrate a time-synthetic ONNs with integrating programmable gain, which can overcome the long-standing limitation of ONN in effective depth and large cumulated loss. The authors provide an ingenious method to compensate the loss by encoding the optical gain into the time-synthetic dimension instead of a spatial photonic mesh, which then avoids the feedback loops and parasitic reflections. This idea is very interesting, and the results should attract many attentions from relevant fields. However, I still have some questions before I can suggest the acceptance.

1. The major issue is how the gain is added into this time-synthetic dimension through PM/MZM. It seems that these two components bring more loss into the system. In addition, in the previous non-Hermitian synthetic constructions, the MZM acts as the loss component [see Nature 598, 59 (2021); Light: Science & Applications 14, 39 (2025) et al.].

2. The authors state that traditional method of integrating gain directly into the computational pathways of an ONN brings unavoidable feedback loops and unwanted parasitic reflections. However, they also put EDFAs in their loops to compensate for MZM-induced suppression loss. Does the gain from EDFAs affect the ONN in time-synthetic dimension? How can the authors eliminate this influence?

3. In the discussion part, the authors give a theoretical area efficiency of 13.6 Tera-FLOPs/mm^2/s for integrated photonic circuit, but did not calculate their efficiency for the proposed fiber loops. Can the authors calculate the processing rate/efficiency for the fiber-based ONNs?

4. What role does nonlinearity in Fig. 1 play? It seems that the authors just covered the topic in the beginning and then didn't elaborate on it further.

5. How does the two PDs monitor the optical power in each loop? How can one to distinguish the beginning and output positions in experiment? And how to obtain Fig. 2b from the experimental data?

Version 1:

Reviewer comments:

Reviewer #1

(Remarks to the Author)
The authors have made considerable efforts to address the concerns raised in the first round of review. The inclusion of the preprocessing details, along with the physical explanations for the distributed encoding and the clarification of the in-situ training dynamics, has significantly enhanced the transparency and readability of the manuscript. Therefore, I recommend publication of the manuscript in Nature Communications.

Reviewer #2

(Remarks to the Author)
The revision addresses the previous comments, and the manuscript is now recommended for publication.

Reviewer #3

(Remarks to the Author)
The authors have successfully addressed all my previous concerns. The manuscript is greatly improved in its quality. I find this work can provide important understanding in the field of ONN with synthetic dimensions.

Open Access This Peer Review File is licensed under a Creative Commons Attribution 4.0 International License, which permits use, sharing, adaptation, distribution and reproduction in any medium or format, as long as you give appropriate credit to the original author(s) and the source, provide a link to the Creative Commons license, and indicate if changes were made.
In cases where reviewers are anonymous, credit should be given to 'Anonymous Referee' and the source.
The images or other third party material in this Peer Review File are included in the article's Creative Commons license, unless indicated otherwise in a credit line to the material. If material is not included in the article's Creative Commons

license and your intended use is not permitted by statutory regulation or exceeds the permitted use, you will need to obtain permission directly from the copyright holder.
To view a copy of this license, visit https://creativecommons.org/licenses/by/4.0/

Response Letter to Reviewers

We really appreciate the valuable comments given by all the reviewers on this manuscript (No. NCOMMS-25-101441).

In the following text, we quote each comment from each reviewer in *italics* and reply one by one. Accordingly, we also revised the main manuscript and Supporting Information while these updates are highlighted in blue. In the text below, the references to these updates are highlighted in a similar way (i.e., highlighted in blue).

GENERAL COMMENTS FROM 1st REVIEWER:

The paper introduces a coupled dual-loop time-synthetic neural network platform, which can perform arbitrary complex-valued linear transformations and effectively reduce the propagation loss. In the system, the programmable gain and phase modulators were utilized to construct a non-Hermitian computational lattice. By separating network depth from the number of physical components, the architecture can simulate over 30,000 effective optical gates in a compact space and achieves remarkable scalability. The experimental results substantiate the critical role of gain, showing that with appropriate gain assistance, the network could attain high accuracy on MNIST classification, whereas without gain, performance collapses remarkably. Furthermore, the authors develop an in-situ training framework that directly derives gradients from measured optical intensities, allowing the system to adapt to hardware imperfections and noise, which enhances robustness and achieves acceptable accuracy on CIFAR-10. These contributions collectively establish gain-assisted time-synthetic ONNs as a stable, scalable, and programmable pathway toward deep photonic intelligence, effectively resolving the tension between gain necessity and instability in spatial photonic systems. The manuscript is compellingly presented through both theoretical analysis and experimental validation and is generally well-structured and clearly written. However, there are still some technical problems and details should be clarified by the authors. I would like to recommend publication of the manuscript after the following issues are addressed.

Response from Authors:

We thank the reviewer for the favourable recommendation for publication of this work in *Nature Communications*, and for commenting that "*These contributions collectively establish gain-assisted time-synthetic ONNs as a stable, scalable, and programmable pathway toward deep photonic intelligence, effectively resolving the tension between gain necessity and instability in spatial photonic systems.*" and "*The manuscript is compellingly presented through both theoretical analysis and experimental validation and is generally well-structured and clearly written.*"

In the following, we fully address the specific comments point-by-point.

SPECIFIC COMMENTS FROM 1st REVIEWER:

1st Reviewer -- Comment 1:

The manuscript employs an unconventional encoding scheme where input data is distributed across the phase modulation parameters of different time layers. Consequently, data encoded in subsequent layers interacts complexly with the signals propagated from previous layers, which appears equivalent to performing a specific pre-processing on the input data. Could the authors clarify whether this distributed encoding strategy provides a unique computational advantage, or is it simply a workaround due to the insufficient number of grid points in a single layer to accommodate all input data? A more explicit explanation is recommended.

Response from Authors:

We thank the reviewer for this insightful comment.

In this work, encoding input data into the phase modulation parameters provides structural nonlinearity to the ONNs, while distributing input data across different time layers is primarily motivated by the insufficient number of grid points in the first time layer.

First, encoding input data into the phase modulation parameters introduces structural nonlinearity to the ONNs [*Nat. Photon.* 18, 1076-1082 (2024); *Nat. Phys.* 20, 1434-1440 (2024)]. Although the field propagation is linear, the mapping from the phase-encoded input to the measured output intensity is nonlinear, i.e., $I(\varphi) = |E(\varphi)|^2$, which contains cross terms among phase variables, such as $\cos(\varphi_i - \varphi_j)$. This nonlinear mapping is essential for ONNs to learn and represent complex decision boundaries.

Second, the distributed encoding strategy is primarily motivated by the insufficient number of grid points in the first time layer. In our experimental implementation, only a single pulse is injected into the coupled loops, and then it gradually spreads to more lattice sites. Although injecting multiple pulses is theoretically feasible, this would introduce initial phase discrepancies. Under this condition, if all input information were encoded only in the phase modulation parameters of the first time layer, a large fraction of the input information would not effectively interact with the propagating optical field, simply because the field has not yet spread to those sites.

Under this physical constraint, we empirically find that distributing input data in time layers L_{12} - L_{17}, L_{20} - L_{21}, and L_{24} - L_{25} gives the best performance among the configurations we tested. To avoid overstatement, we do not claim that this distributed strategy is universally optimal for all architectures. Rather, for the time-synthetic ONN, it provides a physically well-matched and empirically advantageous way of encoding input information into the time-synthetic photonic lattice.

Accordingly, we have added the above encoding details on lines 140-145, page 6 of the main text, which reads,

"This layer-wise encoding is adopted because in our calculation (and experiment shown later) the time-synthetic evolution is initiated from a single pulse, and the optical field gradually

spreads over the temporal lattice as the round-trip proceeds; encoding input images at selected later layers ensures efficient phase modulation to the evolving optical state, so that the encoded information effectively participates in the computation."

<u>1st Reviewer -- Comment 2:</u>

The experimental setup utilizes relatively long optical fiber loops, which are inherently susceptible to environmental perturbations such as acoustic vibrations and thermal drift. Standard experiments involving long fibers typically require rigorous acoustic and thermal isolation, yet such measures appear to be absent in the described setup. Apart from relying on the in-situ training framework for error compensation, did the authors implement signal averaging over multiple measurements during data acquisition to mitigate the stochastic impact of fiber jitter?

<u>Response from Authors:</u>

We thank the reviewer for raising this important point.

We would like to emphasize that our experimental setup follows a standard coupled fiber-loop configuration widely adopted in prior studies for time-synthetic lattice, which has been extensively validated to operate reliably under typical laboratory conditions [*Nature* 488, 167-171 (2012); *Nat. Phys.* 13, 545-550 (2017); *Nature* 601, 354-359 (2022)]. Correspondingly, we implement standard stabilization measures to mitigate acoustic and thermal perturbations, including mechanical fixation of the fiber spools and optical components. Under these standardized conditions, the optical signals are sufficiently stable for data acquisition, consistent with the robustness reported for similar fiber-loop configurations in the literature.

Importantly, our system is designed to be tolerant of moderate environmental fluctuations. The residual perturbations (e.g., slow thermal drift and moderate stochastic phase/polarization jitter) manifest as relatively small deviations on top of the stable signal baseline. On top of this stable baseline, we further employ in-situ training strategy to adapt the model parameters to the actual hardware transfer characteristics and thereby suppress the impact of such residual imperfections, improving the robustness and performance.

We clarify that we do ***not*** implement signal averaging over multiple measurements during data acquisition; all measurements are performed in a single pass. However, to assess their repeatability and stability, we conduct repeated measurements on a representative case, as detailed in our response to comment 5.

Accordingly, we have added the above experimental setup description on lines 277-279, page 11 of the main text, which reads,

"It follows a standard coupled fiber loop configuration widely employed in prior studies for time-synthetic lattice, which has been validated to operate reliably under typical laboratory conditions[20,21,25,34,43-45]."

<u>1st Reviewer -- Comment 3:</u>

*I have concerns regarding the mechanism and validity of the results obtained from the in-situ training algorithm, and I request that the authors provide further clarification on the following details. It is unclear whether the in-situ training algorithm yields a single set of static compensation parameters applied globally during testing, or if it performs real-time dynamic compensation for each instance. If it is the former (static compensation), the results presented in Fig. 4e are perplexing. First, the manuscript lacks sufficient detail regarding the specific 10*10 matrix evaluated in Fig. 4e; it does not explain what this matrix represents or how the "target" and "achieved" matrices were constructed for the fidelity comparison. More critically, Fig. 4e shows that the experimental fidelity after in-situ training (98.5%) significantly exceeds the theoretical in-silico fidelity (94.8%). This is logically counter-intuitive. Theoretically, in-situ training aims to compensate for systematic errors (e.g., fabrication defects) to approach the ideal model. Therefore, the noise-free theoretical model should serve as the upper performance bound, rather than being outperformed by the experiment. If it is the latter (real-time dynamic compensation), this raises a serious concern regarding experimental design. If the algorithm utilizes the ground truth labels of the test data to calculate gradients and adjust parameters on-the-fly during the inference phase, this constitutes data leakage. Please explicitly clarify whether the one-hot encoded ground truth labels were strictly isolated from the algorithm during the inference phase to ensure no data leakage occurred.*

<u>Response from Authors:</u>

We thank the reviewer for the careful comments. In the following, we explicitly clarify five points: (1) the distinction between in-silico and in-situ training, (2) whether any data leakage occurs in the image-classification experiments, (3) the 10×10 matrix representation, (4) the construction of “target” and “achieved” matrices, and (5) the comparison between in-silico and in-situ fidelity.

First, we clarify the distinction between in-silico and in-situ training. In our manuscript, in-silico training refers to optimizing the network parameters in an ideal theoretical model and then directly mapping the optimized parameters onto the imperfect physical system. By contrast, in-situ training refers to optimizing the parameters using experimentally measured optical signals, to compensate for simulation-to-hardware mismatch and hardware imperfections.

Second, we clarify that there is ***no*** data leakage in the image-classification experiments. During training, the model yields a single set of static compensation parameters (i.e., static compensation): once trained, the parameters are applied globally during testing. ***No*** real-time dynamic compensation is performed for each instance. Therefore, the one-hot encoded ground truth labels are strictly isolated from the algorithm during the inference phase, and ***no*** data leakage occurred.

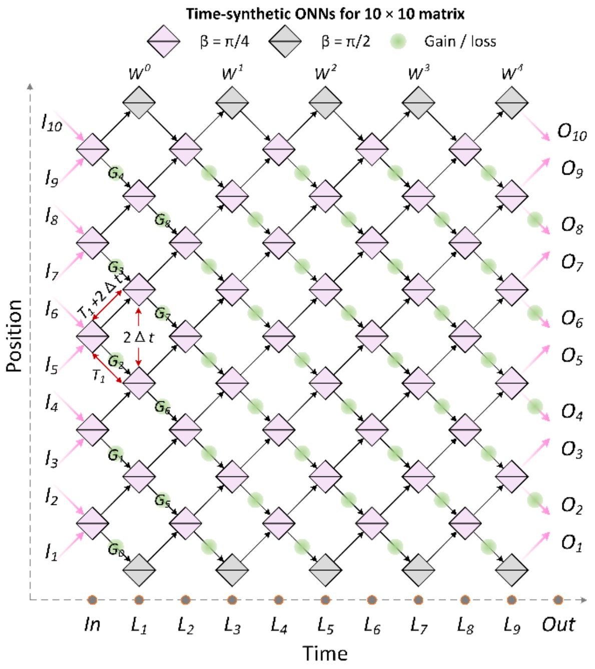

Fig. R1 | The time-synthetic ONNs for 10×10 matrix representation. The splitting ratio at the boundary is set to $\beta = \pi/2$, such that the pulses are totally reflected.

Third, we clarify the 10×10 matrix representation. The 10×10 matrix represents the linear transformation to be implemented by the time-synthetic ONNs, as shown in Fig. R1. The input-output relation is given by $O = TI$, where I and O are the complex amplitude vectors of the ten input and the ten output pulses, respectively. According to the modified discrete quantum walk equations that govern pulse propagation, the matrix T is a function of the gain/loss array:

$$T = W^0 W^1 W^2 W^3 W^4 \tag{1}$$

$$W^n = \begin{bmatrix} -G_0^n/\sqrt{2} & iG_0^n/\sqrt{2} & 0 & 0 & 0 & 0 & 0 & 0 & 0 & 0 \\ iG_5^n/2 & -G_5^n/2 & iG_1^nG_5^n/2 & G_1^nG_5^n/2 & 0 & 0 & 0 & 0 & 0 & 0 \\ 1/2 & i/2 & -G_1^n/2 & iG_1^n/2 & 0 & 0 & 0 & 0 & 0 & 0 \\ 0 & 0 & iG_6^n/2 & -G_6^n/2 & iG_2^nG_6^n/2 & G_2^nG_6^n/2 & 0 & 0 & 0 & 0 \\ 0 & 0 & 1/2 & i/2 & -G_2^n/2 & iG_2^n/2 & 0 & 0 & 0 & 0 \\ 0 & 0 & 0 & 0 & iG_7^n/2 & -G_7^n/2 & iG_3^nG_7^n/2 & G_3^nG_7^n/2 & 0 & 0 \\ 0 & 0 & 0 & 0 & 1/2 & i/2 & -G_3^n/2 & iG_3^n/2 & 0 & 0 \\ 0 & 0 & 0 & 0 & 0 & 0 & iG_8^n/2 & -G_8^n/2 & iG_4^nG_8^n/2 & G_4^nG_8^n/2 \\ 0 & 0 & 0 & 0 & 0 & 0 & 1/2 & i/2 & -G_4^n/2 & iG_4^n/2 \\ 0 & 0 & 0 & 0 & 0 & 0 & 0 & 0 & i/\sqrt{2} & -1/\sqrt{2} \end{bmatrix} \tag{2}$$

Fourth, we clarify the construction of "target" and "achieved" matrices. To compare the in-silico and in-situ fidelity, we randomly generated different gain/loss configurations. For each configuration, the target matrix is calculated using the ideal theoretical model (Eqs. 1-2). To obtain the experimentally realized matrices, the same gain/loss configurations are applied to the physical system. Unit-amplitude pulses are injected one at a time into the ten input ports (with the other nine ports receiving zero input), and the measured complex amplitudes at the ten output ports form the columns of the realized matrix. This procedure is repeated for all

randomly generated gain/loss configurations, and the resulting fidelity yields the value reported in Fig. 4e.

Finally, we compare the in-silico and in-situ fidelity of the 10×10 matrix. The reviewer points out that the in-situ fidelity (98.5%) exceeds the in-silico fidelity (94.8%). We emphasize that the 94.8% value does ***not*** correspond to a noise-free ideal upper bound. Instead, it corresponds to the experimental implementation when directly mapping ideal parameters onto the hardware, which is limited by the unavoidable discrepancies between the ideal theoretical model and the actual physical system. In contrast, in-situ training fine-tunes parameters to approach the ideal output and therefore can achieve a higher fidelity.

We appreciate the reviewer's suggestion that a detailed description would be helpful to readers. Accordingly, we have added a new section "Implementation of 10 × 10 matrix" in Supplementary Note 2.

We also added a sentence referring to Supplementary Note 2 on lines 228-229, page 9 of the main text, which reads,
"The in-situ training framework can compensate for the calibration drift to some extent, and we evaluate its effectiveness by performing 10×10 matrix operations (detailed in Supplementary Note 2)."

We also clarified the definition of in-silico training on line 190, page 8 of the main text, which reads,
"Unlike in-silico training, which deploys theoretical parameters on the imperfect photonic hardware without any calibration […]".

1st Reviewer -- Comment 4:
I have concerns regarding the proposed neural network architecture and its reported performance. Drawing an analogy to electronic neural networks, if each lattice point is treated as a neuron that couples only with two adjacent points in the subsequent time layer, the proposed architecture essentially functions as an extremely sparsely connected fully connected network (FCN). Theoretically, the computational capacity of such an architecture should be limited. Similar to standard FCNs, this approach appears to process images as flattened inputs, implying it lacks the inherent capability of Convolutional Neural Networks (CNNs) to effectively capture local spatial features. However, the manuscript reports an accuracy of 86.5% on the CIFAR-10 dataset, remarkably achieved after applying grayscale conversion and cropping—preprocessing steps that typically result in information loss and reduced accuracy. This result appears anomalous, as it significantly outperforms not only standard CNN benchmarks for CIFAR-10 (approximately 82%) but also certain optimized deep convolutional architectures (e.g., 84.87% reported in Stochastic Pooling for Regularization of Deep Convolutional Neural Networks). Given the structural sparsity of the network and the lossy preprocessing of the input data, such superior performance seems counter-intuitive. I request that the authors provide a rigorous explanation for this discrepancy.

Response from Authors:

We thank the reviewer for the insightful comment.

We agree that, if only considering the coupling between two adjacent time layers, the connectivity of the proposed architecture appears highly local and sparse. However, the influence of each input feature progressively spreads over an expanding region of the lattice as the signal propagates, resulting in an ***almost fully connected*** input-output transformation. This depth-enabled full connectivity is conceptually analogous to MZI meshes in integrated photonics circuits, where cascaded local interferometric operations collectively realize general matrix transformations [*Nat. Photon.* 11, 441-446 (2017); *Nature* 640, 368-374 (2025)].

With this perspective, our architecture is closer to a deep globally-mixing photonic processor rather than a conventional model with local receptive fields. Accordingly, the reported accuracy should ***not*** be directly compared with results achieved by CNNs, whose performance relies on different inductive biases. Importantly, the state-of-the-art spatial ONN architecture has already achieved an accuracy of 93.7% on CIFAR-10 dataset [*Science* 384, 202-209 (2024)]. In this context, our reported accuracy lies within a reasonable range when sufficient network depth, structural nonlinearity, and feature engineering pipeline are taken into account.

Accordingly, we have added the above network connectivity description on lines 115-117, page 5 of the main text, which reads,
"Although each weight matrix $W(G^n, \varphi^n)$ is sparse, the product of multiple weight matrices becomes dense, resulting in an almost fully connected input-output transformation."

1st Reviewer -- Comment 5:

Although the authors introduce optical gain to compensate for propagation loss, various components within the loops—such as PM, MZM, and EDFAs—inevitably introduce irregular noise, which accumulates with the number of round trips. The manuscript does not provide a quantitative analysis of this accumulated noise, nor does it clearly specify the upper limit on the number of round trips over which optical pulses can maintain sufficiently high fidelity for reliable computation.

Response from Authors:

We thank the reviewer for this constructive suggestion.

We agree that although optical gain compensates for propagation loss, the components in the loops inevitably introduce irregular noise, which accumulates with the number of round trips. To quantitatively analyse the impact of noise on fidelity, we conduct additional experiments using the same configuration as in the image classification tasks. Specifically, a square pulse with >50 dB extinction ratio and 50 ns temporal width is injected into the longer loop. The variable BS splits each pulse with a predefined beam-splitting ratio of 50:50, and the shorter loop integrates an MZM for dynamic gain/loss modulation.

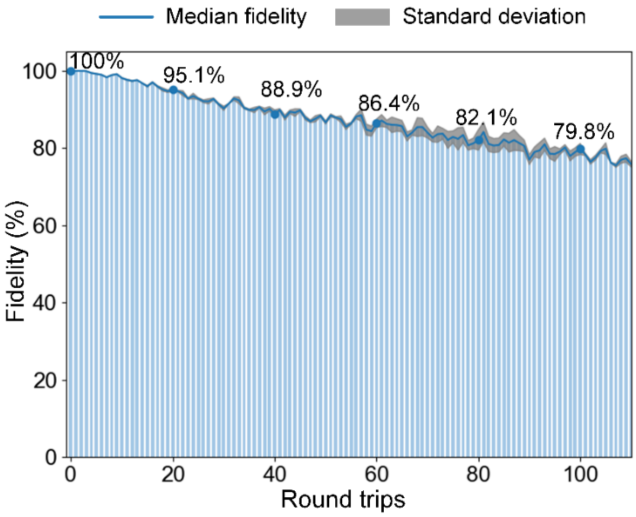

Fig. R2 | The fidelity at different round trips.

To evaluate the repeatability and stability of the measurements, we perform repeated measurements under a fixed gain/loss configuration and calculate the fidelity for up to 110 round trips using the formula $F = (1 - |\hat{Y} - Y|/(\hat{Y} + Y)) \times 100\%$, where Y is the optical signals without distortion at each round trip, and $\hat{Y}$ is the experimentally measured optical signals.

The measured results are shown in Fig. R2. The blue solid line denotes the median across repeated measurements, and the shaded region denotes the standard deviation. The experimental results show that the fidelity gradually decreases with the number of round trips, but remains sufficiently high (88.9%) over the range used in our experiments (**40** round trips). After **110** round trips, the fidelity remains at 75.9%, which exceeds a commonly used engineering threshold of 67% (corresponding to an SNR of 3 dB), indicating that the time-synthetic ONN can still operate effectively with the assistance of in-situ training.

Accordingly, we have added a new section “Quantitative analysis of fidelity” in Supplementary Note 1.

We also added a sentence referring to Supplementary Note 1 on lines 167-168, page 7 of the main text, which reads,
“More details about the experimental noise analysis can be found in Supplementary Note 1.”

<u>1st Reviewer -- Comment 6:</u>

In lines 226–231, the effective number of gates is estimated based on the evolution of a single pulse in the synthetic temporal lattice. However, since the number of pulses increases with each round trip, the total number of effective gates cannot be simply expressed as the product of the number of pulses accommodated by the fiber and the number of round trips. A more rigorous justification or clarification of this scaling relation is required.

<u>Response from Authors:</u>

We thank the reviewer for raising this important point.

We agree that, in our time-synthetic lattice, the number of pulses increases with each round trip, and the total number of effective gates cannot be simply expressed as the product of the number of pulses accommodated by the fiber and the number of round trips.

In our experiments, we operate the system in a proof-of-concept manner and inject only a single pulse into the coupled loops to enable a reliable demonstration and characterization. The "effective number of gates" in lines 226-231 is intended to describe an in-principle capacity under a fully-loaded operation, where one can simultaneously excite all lattice sites in the first time layer, as detailed in Supplementary Note 5. In this case, the number of effective gates is designed to be approximately constant for each round trip, and the effective number of gates scales with the number of populated lattice sites and the number of round trips.

Following the reviewer's suggestion and to avoid any potential confusion, we have specified the constraints for the effective gate number on lines 238-239, page 10 of the main text, which reads,
"[…]generating 31,124 effective gates in a fully loaded propagation scheme (detailed in Supplementary Note 5)".

1st Reviewer -- Comment 7:
The authors mention that the system remains stable as long as the programmed gain stays below the point where instability would occur. They should provide a detailed explanation of the theoretical methods or experimental approaches used to determine this instability threshold in synthetic temporal lattice.

Response from Authors:
We thank the reviewer for raising this important point.

In our system, "instability" refers to the onset of self-amplifying growth of the circulating optical energy across round trips, manifested experimentally as a rapid increase or oscillatory divergence of the pulse energy. To determine the instability threshold, we perform repeated gain sweeps and monitor the round-trip evolution of the circulating pulse energy. Empirically, we find that the system remains stable when the excess of the externally programmed average gain over loss satisfies $\langle \ln(G/L) \rangle \lesssim 0.1$. In this regime, the effective gain does not exceed the loss (including both intrinsic loss and externally programmed loss).

To maintain the system stable, we adopt a two-stage training protocol. First, the ONN is pre-trained on a theoretical model to capture the ideal propagation dynamics. In this stage, the input consists of a unit-amplitude pulse, and the ground-truth is one-hot encoded; consequently, the trained parameters do not drive the system into net amplification. Second, the pre-trained model is fine-tuned on the physical hardware to adapt to noise and hardware imperfections. In this stage, we use a small learning rate to prevent an abrupt increase in gain factors that could drive the system into oscillation.

Accordingly, we have added a new section "Stability conditions and training protocol" in Method.

<u>1st Reviewer -- Comment 8:</u>

With hundreds of pulses propagating concurrently in the loops, potential crosstalk between adjacent pulses could affect computational accuracy. The paper reports a coupled experimental platform containing over 30,000 effective time gates. What experimental or analytical evidence proves that the computational precision (e.g., the effective bit-depth or phase stability of the artificial neurons) does not degrade after, for example, 100 round trips? Furthermore, are there error correction or calibration routines embedded in the in-situ training that specifically compensate for predictable distortion accumulated over time?

<u>Response from Authors:</u>

We thank the reviewer for this good question.

Regarding inter-pulse crosstalk, although many pulses propagate concurrently in the coupled loops, they are well separated in time. Specifically, the pulse duration is ~50 ns, and the minimum pulse-to-pulse spacing is ~196 ns. This provides a substantial temporal guard interval (several pulse widths) between adjacent pulses, strongly suppressing inter-pulse crosstalk. Consistent with this, the measured time-series optical signals do not show noticeable leakage from one pulse into neighboring pulses under our operating conditions.

Regarding computational precision after 100 round trips, we evaluate fidelity as a function of round-trip count, as detailed in our response to comment 5. After **100** round trips, the fidelity remains at 79.8%, surpassing the engineering detection threshold of 67% (corresponding to an SNR of 3 dB). This indicates that the time-synthetic ONN remains capable of effective operation when supported by in-situ training. This depth-dependent fidelity provides a direct experimental proxy for the effective computational precision at large round-trip counts, and it indicates no abrupt collapse up to 100 round trips in our system.

Regarding error correction or calibration routines, the in-situ training does ***not*** embed a separate calibration algorithm. Instead, predictable distortions and systematic hardware imperfections are implicitly compensated through the in-situ training procedure: by optimizing the network parameters with experimentally measured optical signals, the trained parameters learn the hardware-specific transfer characteristics and reduce the impact of repeatable distortions accumulated over round trips [*Nature* 632, 280-286 (2024)]. The parameters are then fixed during testing, ensuring no real-time dynamic compensation is performed for each instance.

Accordingly, we have added the above crosstalk analysis on lines 86-87, page 4 of the main text, which reads,

"In our experiment, the pulse width is 50 ns, and the temporal offset $2\Delta t$ = 196 ns, which is designed to suppress the inter-pulse crosstalk."

GENERAL COMMENTS FROM 2nd REVIEWER:

This work presents an experimental demonstration of optical neural networks based on coupled fiber loops, demonstrating a benefit of optical gain on the network operation. Such experimental systems, also incorporating gain elements, have been introduced and extensively investigated for over a decade. The main novelty here is the analysis of gain effect specifically for neural-network applications.

Overall, the technical quality is high, and results are generally convincing. There are some questions that need clarification:

Response from Authors:

We thank the reviewer for the favourable recommendation for publication of this work in *Nature Communications*, and for commenting that "*Overall, the technical quality is high, and results are generally convincing.*"

In the following, we fully address the specific comments point-by-point.

SPECIFIC COMMENTS FROM 2nd REVIEWER:

2nd Reviewer -- Comment 1:

The aspect of phase-modulation is not fully clear. The theoretical design incorporates the training of nontrivial phase modulation. However, in experiments, the phase modulation is absent, in order to perform experimental training just based on the output intensities. Then, why was the phase modulation included in the theory at all? Is there any specific downside to omitting the phase modulation in experiments?

Response from Authors:

We thank the reviewer for raising this important point.

Phase modulation is included in the theoretical model because it represents a degree of freedom of the time gate. As shown in Fig. 1c in the main text, each time gate independently modulates gain/loss and phase shifter, which reflect the full capacity of the time gate.

In our experimental setup, we also incorporate a phase modulator to support the phase shifter. For experimental convenience in the proof-of-concept demonstration, we disable phase modulation and optimize only gain/loss factors, which allows in-situ training without phase recovery. This choice sacrifices one learnable degree of freedom for time-synthetic ONNs.

We note that this limitation is ***not*** fundamental: phase can be obtained by introducing phase-sensitive measurement schemes [*Nat. Commun.* 15, 6339 (2024)]. With such measurements, phase modulation can be fully utilized in experiments, which we plan to pursue in follow-up implementations.

Accordingly, we have added a description of this trade-off on lines 193-194 and 200-201, page 8 of the main text, which reads,

“For experimental convenience in this proof-of-concept demonstration, we disable phase modulation and optimize only gain/loss factors, which allows in-situ training without phase recovery. […] While optical phase is not measured in the present work, it could be probed in future implementations via phase-sensitive readout.”

2nd Reviewer -- Comment 2:

Is there a practical limit on the number of round-trip times. It is about 40 in the current study, but what is a practical limit, and how does the gain and noise influence it?

Response from Authors:

We thank the reviewer for this good question.

The practical limit is primarily determined by the trade-off between optical gain and accumulated noise. Optical gain compensates for propagation loss and prevents the signal from decaying below the electronic noise floor of the PD, but also introduces accumulated noise.

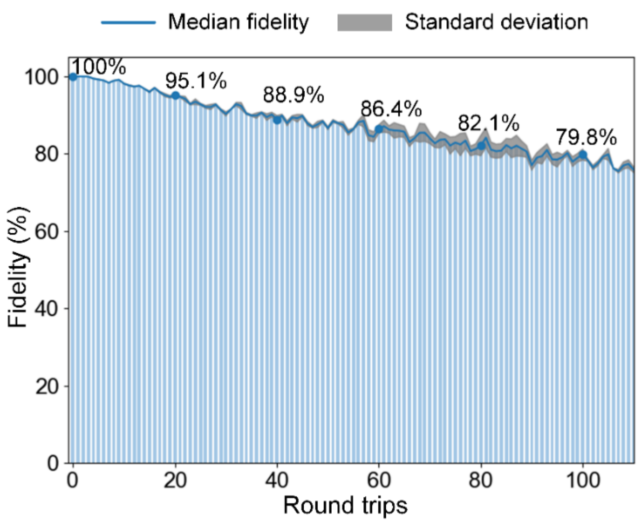

Fig. R3 | The fidelity at different round trips.

To quantitatively analyse the impact of noise on fidelity, we conduct additional experiments using the same configuration as in the image classification tasks. Specifically, a square pulse with >50 dB extinction ratio and 50 ns temporal width is injected into the longer loop. The variable BS splits each pulse with a predefined beam-splitting ratio of 50:50, and the shorter loop integrates an MZM for dynamic gain/loss modulation.

To evaluate the repeatability and stability of the measurements, we perform repeated measurements under a fixed gain/loss configuration and calculate the fidelity for up to 110 round trips using the formula $F = (1 - |\hat{Y} - Y|/(\hat{Y} + Y)) \times 100\%$, where Y is the optical signals without distortion at each round trip, and $\hat{Y}$ is the experimentally measured optical signals.

The measured results are shown in Fig. R3. The blue solid line denotes the median across repeated measurements, and the shaded region denotes the standard deviation. The

experimental results show that the fidelity gradually decreases with the number of round trips, but remains sufficiently high (88.9%) over the range used in our experiments (**40** round trips). After **110** round trips, the fidelity remains at 75.9%, which exceeds a commonly used engineering threshold of 67% (corresponding to an SNR of 3dB), indicating that the time-synthetic ONN can still operate effectively with the assistance of in-situ training.

Accordingly, we have added a new section "Quantitative analysis of fidelity" in Supplementary Note 1.

We also added a sentence referring to Supplementary Note 1 on lines 167-168, page 7 of the main text, which reads,
"More details about the experimental noise analysis can be found in Supplementary Note 1."

GENERAL COMMENTS FROM 3rd REVIEWER:
Wu et al. demonstrate a time-synthetic ONNs with integrating programmable gain, which can overcome the long-standing limitation of ONN in effective depth and large cumulated loss. The authors provide an ingenious method to compensate the loss by encoding the optical gain into the time-synthetic dimension instead of a spatial photonic mesh, which then avoids the feedback loops and parasitic reflections. This idea is very interesting, and the results should attract many attentions from relevant fields. However, I still have some questions before I can suggest the acceptance.

Response from Authors:
We thank the reviewer for the favourable recommendation for publication of this work in *Nature Communications*, and for commenting that "*This idea is very interesting, and the results should attract many attentions from relevant fields.*"

In the following, we fully address the specific comments point-by-point.

SPECIFIC COMMENTS FROM 3rd REVIEWER:
3rd Reviewer -- Comment 1:
The major issue is how the gain is added into this time-synthetic dimension through PM/MZM. It seems that these two components bring more loss into the system. In addition, in the previous non-Hermitian synthetic constructions, the MZM acts as the loss component [see Nature 598, 59 (2021); Light: Science & Applications 14, 39 (2025) et al.].

Response from Authors:
We thank the reviewer for raising this important point.

The reviewer is right that "*the MZM acts as the loss component*". In our platform, the key to achieving programmable gain/loss is the co-modulation of MZM and EDFAs, rather than the MZM alone.

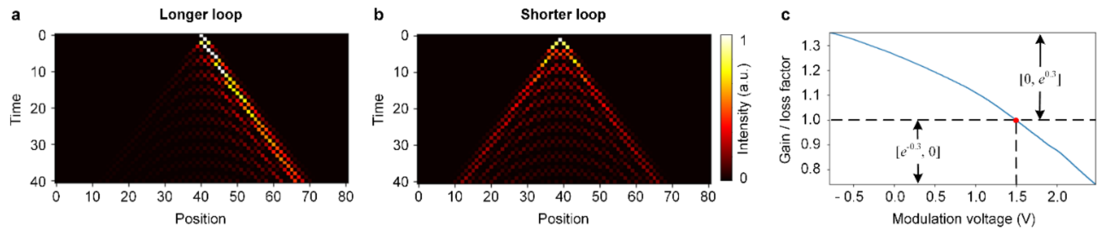

Fig. R4 | The propagation field in **a,** the longer and **b,** the shorter loop in a unity-gain system. **c,** The curve of the gain/loss factor as a function of the MZM modulation voltage, where 1.5 V corresponds to the unity-gain state.

We first set the MZM modulation voltage to 1.5 V and then adjust the gains of EDFAs in the longer and the shorter loops until the measured propagation fields match the theoretical prediction for a unity-gain system, as shown in Fig. R4a and R4b. This calibration ensures that, at 1.5 V the total loss is balanced by the EDFA gain.

After the unity-gain baseline is established, we sweep the MZM modulation voltage and measure the output amplitude relative to the unity-gain state. As shown in Fig. R4c, this procedure allows us to span the target gain/loss range of $\left[e^{-0.3}, e^{0.3}\right]$ used in our experiments. In this way, the gain regime is realized when the MZM loss is reduced relative to the reference state, and the loss regime is realized when the MZM loss is increased relative to that state.

Accordingly, we have added a new section "Implementation of programmable gain/loss" in Supplementary Note 3.

We have also cited the references in the revised manuscript, as shown below:
"Each loop contains an erbium-doped fiber amplifier (EDFA) to compensate for MZM-induced suppression loss and passive damping effects[43,44].

43. Wang, K., et al. Topological complex-energy braiding of non-Hermitian bands. *Nature* **598**, 59-64 (2021).
44. Ye, R., et al. Observing non-Hermiticity induced chirality breaking in a synthetic Hall ladder. *Light: Sci. Appl.* **14**, 39 (2025)."

3rd Reviewer -- Comment 2:

The authors state that traditional method of integrating gain directly into the computational pathways of an ONN brings unavoidable feedback loops and unwanted parasitic reflections. However, they also put EDFAs in their loops to compensate for MZM-induced suppression loss. Does the gain from EDFAs affect the ONN in time-synthetic dimension? How can the authors eliminate this influence?

Response from Authors:

We thank the reviewer for this insightful question.

We agree that any amplifier can bring feedback loops and unwanted parasitic reflections. In spatial networks, multiple optical pathways make these issues hard to avoid, and once gain is

introduced, self-oscillation can easily occur. By contrast, our time-synthetic ONN computes through a causal, forward evolution along the time synthetic dimension: signals propagate step-by-step to subsequent time layers, rather than propagating through multiple optical pathways.

Consequently, compared to spatial networks, our architecture makes feedback much easier to control. It reduces to suppressing backward propagation in two predefined loops instead of eliminating numerous internal feedback paths distributed throughout a mesh. In practice, only two optical isolators can prevent the parasitic reflections in the coupled loops that would disrupt the intended time-synthetic computation.

Accordingly, we have added a new section "Elimination of parasitic reflections" in Supplementary Note 4

3rd Reviewer -- Comment 3:

In the discussion part, the authors give a theoretical area efficiency of 13.6 Tera-FLOPs/mm^2/s for integrated photonic circuit, but did not calculate their efficiency for the proposed fiber loops. Can the authors calculate the processing rate/efficiency for the fiber-based ONNs?

Response from Authors:

We thank the reviewer for raising this important point.

For the current fiber-based ONN, area efficiency is not a meaningful metric, because the physical footprint is dominated by discrete optical components rather than by an area-optimized on-chip layout. Instead, for the present fiber-loop system, the operational speed is a more appropriate metric. In our current experimental setup, the resulting operational speed is about $R = (6T/\Delta t - 3)/2T = 30.55$ MOPS.

Importantly, we did ***not*** maximize the operational speed in the current experimental setup. The pulse-to-pulse spacing $2\Delta t$ is chosen conservatively to ensure robust operation and reliable characterization. With an optimized implementation (e.g., reducing $2\Delta t$ while maintaining stable modulation and detection), the operational speed can be increased by approximately an order of magnitude.

Looking forward, the operational speed can be further increased in two straightforward directions. First, the pulse-to-pulse spacing $2\Delta t$ is fundamentally constrained by the sampling rate of the AWGs, which determines the pulse modulation speed. Our current AWGs operate at 1 GSa/s; upgrading to the Keysight M8194A (120 GSa/s) would increase the operational speed by over two orders of magnitude. Second, leveraging mature parallelization technologies from optical communications, such as wavelength division multiplexing (WDM), can provide near-linear scaling of the operational speed with the number of parallel channels (e.g., ~100× with ~100 WDM channels).

Accordingly, we have added the detailed calculation process of operational speed in Supplementary Note 6.

<u>3rd Reviewer -- Comment 4:</u>

What role does nonlinearity in Fig. 1 play? It seems that the authors just covered the topic in the beginning and then didn't elaborate on it further.

<u>Response from Authors:</u>

We thank the reviewer for this insightful suggestion.

In Fig. 1, the "nonlinearity" refers to the structural nonlinearity exploited in this work. As discussed in the manuscript, although the propagating operator is linear, the output depends nonlinearly on the encoded input parameters, which yields the nonlinear activation function in this linear system. Specifically, encoding the input signals onto the phase shifters produces a complex exponential nonlinearity, whereas encoding the input signals onto the gain/loss factors with duplication produces a polynomial nonlinearity. Such nonlinearity is essential for learning and representing nonlinear input-output mappings; without it, a deep linear system would collapse to an overall linear transformation.

In the digit classification task, the input images are encoded onto the phase shifters, which corresponds to the complex exponential nonlinearity. In the object recognition task, the input images are duplicated and encoded onto the gain/loss factors, which correspond to the polynomial nonlinearity.

Accordingly, we have added the above nonlinearity implementations on lines 140, page 6; lines 208-209, page 8 of the main text, which reads,

"[…] are encoded onto the phase shifters of optical pulses in time layers L_{12} - L_{17}, L_{20} - L_{21}, and L_{24} - L_{25} to introduce the complex exponential nonlinearity."

"[…] encoded into gain/loss factors at designated time layers to introduce the polynomial nonlinearity."

<u>3rd Reviewer -- Comment 5:</u>

How does the two PDs monitor the optical power in each loop? How can one to distinguish the beginning and output positions in experiment? And how to obtain Fig. 2b from the experimental data?

<u>Response from Authors:</u>

We thank the reviewer for this good question.

First, the optical signals in the two loops are monitored by two PDs through 50:50 BSs. As illustrated by red lines in Fig. R5a, each BS taps half of the optical power from the loop and sends it to a PD. The PDs convert the optical signals into electronic signals, which are recorded by a DSO for further analysis.

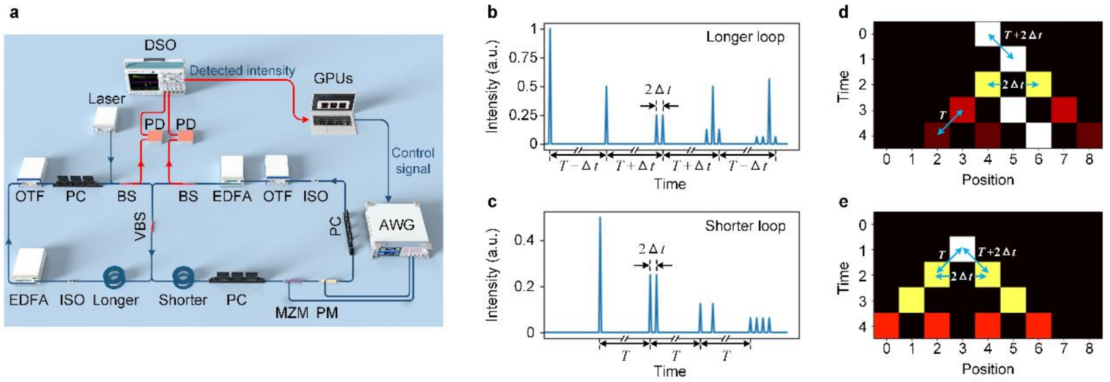

Fig. R5 | a, The optical-loop experimental setup. Taking propagating across four round trips as an example, the time-series optical signals in **b,** the longer loop and **c,** the shorter loop. The propagation fields in **d,** the longer loop and **e,** the shorter loop.

Second, in the monitored time-series signal, the pulse with the highest intensity is taken as the beginning position, denoted by T_1. For a pulse propagating across N round trips, the corresponding output position is $T_1 + N(T + 2\Delta t)$, where T = 24,636 ns is the shorter-loop period and $2\Delta t$ = 196 ns is the temporal separation between the shorter and longer loops.

Third, the propagation field is reconstructed from the measured time-series signals. Specifically, after identifying the beginning and output positions, we extract the useful positions of the time-series signal of the longer and shorter loops; see Fig. R5b and R5c for a 4-round-trip example. Using the known temporal relations (e.g., the shorter-loop period T and the temporal separation $2\Delta t$), the detected time-series signals are mapped onto the time-synthetic lattices. In the reconstructed propagation field, each point corresponds to a pulse, with its lower-left and lower-right neighbors separated by T and $T + 2\Delta t$, respectively, and its horizontal neighbors separated by $2\Delta t$. This procedure yields the propagation field shown in Fig. R5d and R5e.

Accordingly, we have added a new section "Experimental signal monitoring and propagation-field reconstruction" in Supplementary Note 8.
